# Evaluation of seed nitrate assimilation and stimulation of phenolic-linked antioxidant on pentose phosphate pathway and nitrate reduction in three feed-plant species

**DOI:** 10.1186/s12870-020-02453-w

**Published:** 2020-06-09

**Authors:** Derong Lin, Yichen Huang, Jingjing Zhao, Zhijun Wu, Shuliang Liu, Wen Qin, Dingtao Wu, Hong Chen, Qing Zhang

**Affiliations:** 1 School of Food Science, Sichuan Agricultural University, Ya’an, 625014 China; 2 College of Mechanical and Electrical Engineering, Sichuan Agricultural University, Ya’an, 625014 China

**Keywords:** Antioxidant enzymes, Nitrate reductase, Oxidative stress, Phenolic, Proline, Superoxide dismutase

## Abstract

**Background:**

Soil and water pollution due to nitrate are becoming increasingly serious worldwide. The government also put forward relevant governance policies, and a large number of scholars studied chemical physics and other methods to remove nitrate in water, but the cost was substantial. Studies have found that planting systems including grasses have the potential to remove nitrates. However, there are few studies on nitrate linked pathway and nitrate assimilation during its early growth.

**Results:**

We have evaluated three different feed-plant species with three levels of overnight seed nitrate treatments along with a control. The activity of different enzymes from 2 weeks old shoots was measured to get a comprehension of proline-associated pentose phosphate pathway coupled with nitrate assimilation and phenolic-linked antioxidant response system in these species under nitrate treatments. All three feed-plant species showed high nitrate tolerance during germination and early growth stages. It is perceived that the accumulation of total soluble phenolics and total antioxidant activity was high in all three feed-plant species under high nitrate treatments. In terms of high G6PDH activity along with low SDH activity in alfalfa, there may be a shift of carbon flux in this species under high nitrate treatments. Higher activity of these enzymes along with higher SOD and GPX activity was observed in alfalfa. The efficient mechanism of nitrate stress tolerance of alfalfa also correlated with higher photochemical efficiency. Perennial ryegrass also showed excellent potential under high nitrate treatments by adopting an efficient mechanism to counter nitrate-induced oxidative stress.

**Conclusions:**

Under the condition of nitrate treatment, the germination rates of the three feed-plant species are still ideal, and they have good enzyme activity and have the potential to remove nitrate.

## Background

In the Twenty-First Century,as the global population is rapidly increasing, supply of safe drinking water becomes a major challenge. Two-fifths of the population suffers poor sanitary conditions and one-fifths can not have access to safe drinking water in the world [1]. The major sources of ground water contamination are from domestic, industrial and agricultural utilization of renewable fresh water with numerous synthetic, inorganic and geogenic compounds [2]. Nitrate contamination in groundwater originates in nitrogen manure, sewage irrigation, organic manure and livestock farming. A general investigation of different pollutants finds that nitrate (NO_3_¯) is regarded as the most widespread groundwater contaminant in the world. In past decades, a large rise in nitrate concentration of groundwater has happened to many developing and developed countries [3]. To improve crop output per unit area and the general farm products for the requirement of progressively increasing population, a mass of fertilizer is spread on the soil. Nitrogen (N) is regarded as an indispensable investment which determines crop productivity and output in soil [4]. Every human eats almost 4.5 kg of N every year by ingestion of protein. According to statistics, the present world population expands about 28 mt of protein-N per annum [5].

The Environmental Protection Agency (EPA) of the United States has formulated that a maximum contaminant level (MCL) of nitrate in drinking water can be 0.71 mM (10 ppm = 10 mg of NO_3_¯ -N liter^− 1^). High nitrate concentration in groundwater can possibly lead to the formation of N- nitroso compounds which are known to be a carcinogen in the digestion system and may cause potential health risk like methemoglobinemia (blue baby syndrome), especially in infants [6]. In addition, nitrate accumulation in forage crops can also cause nitrate poisoning in ruminants [7]. When nitrate pollutes groundwater, the diversity of aquatic plants involved will be reduced [8]. Zhang et al. found that about 52% of groundwater samples in 69 survey sites in the North China Plain exceeded the allowable limit of nitrate in drinking water [9]. Currell et al. recorded a groundwater sample with a depth of 180 m in Yuncheng Basin, in which the concentration of nitrate nitrogen exceeded 45 mg / L [10]. Among the groundwater samples collected from more than 2000 shallow groundwater monitoring wells in the northern basin of China, 80% of the main pollutants contain nitrate. In addition, in many shallow and deep groundwater systems, as well as in karst landforms, it is found that the median concentration of nitrate-nitrogen exceeds the maximum persistent concentration. The most seriously affected area is the coastal area adjacent to the Bohai Sea [11]. We are aware of the fact that the current situation on nitrate removal concentrates on chemical, physical and biological strategies, but many of them are complex and expensive. On the contrary, plant-based system with high nitrate tolerant plants could be an effective strategy both in the greenhouse and in field or wasteland situations, which has a very big gap with other fields but a great application prospect nowadays. The wetlands planted with different robust plant species behaved high nitrogen pollution removal ability compared to unplanted wetlands [12]. Grass catch crops also diminish N mineralization and the most important is an effective reduction in nitrate leaching [13]. The key to formulate an effective strategy for nitrate removal is the selection of suitable feed-plant species and cultivars through screening.

**Fig. 1:**
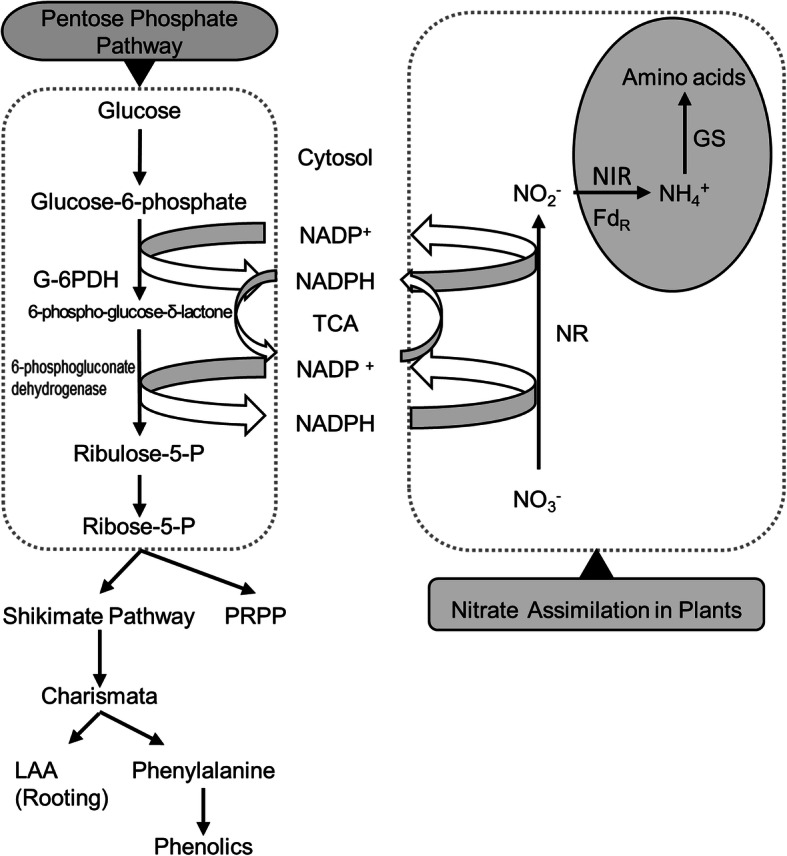
The relation of the enzymes with Nitrogen assimilation and pentose phosphate pathway

It is important to understand the biochemical mechanism for nitrate uptake and assimilation including different pathway regulations in these plants. Nitrate uptake in plants is a protein-mediated process and assimilation of nitrate requires three enzyme-dependent conversions. The process was shown in Fig. 1. Driving pentose phosphate pathway can provide energy (NADPH) for nitrate assimilation and provide growth regulators and phenols needed by plants. Enzymes are essential catalysts for these processes, such as SDH which promotes the production of NADPH in the TCA process. Firstly nitrate (NO_3_¯) is reduced to nitrite (NO_2_¯) by the nitrate reductase (NR), next, the nitrite (NO_2_¯) is converted to ammonium (NH_4_^+^) by nitrite reductase (NIR), and lastly, ammonium is reduced into amino acids with glutamine synthatase/glutamate synthase [14]. The efficient utilization of absorbed nitrate in plants largely relies on the efficiency of reducing nitrate to ammonium and ammonium into amino acids [15]. In the synthesis of nitrate reductase, light is the most important factor in regulating the supply of reductant in this process. Many studies reported that NADPH produced by the oxidative pentose phosphate pathway could act as an alternative to reducing equivalent for nitrate reduction in dark [16–18]. Electrons from NADPH must be found to reduce Fd, which act as electron donor to nitrate reductase. Onset of nitrate (NO_3_¯) assimilation is in accordance with the Fd-thioredoxin-dependent activation of glucose-6-phosphate dehydrogenase (G6PDH), the regulatory enzyme of the oxidative pentose phosphate pathway [16, 19]. Oxidation of carbohydrate through the oxidative pentose phosphate pathway also gives reducing power for nitrite (NO_2_¯) reduction [20, 21].

Pentose phosphate pathway can generate NADPH,which can be utilized for nitrate reduction in the cytosol. The conversion to ribulose - 5 - phosphate along with generation of NADPH by G6PDH is the first committed step of pentose phosphate pathway [22]. Pentose phosphate pathway acted on the shikimate and phenylpropanoid pathways, accumulated phenolic phytochemicals in plants by direct generation or regulatory of the pathway [22–24]. Proline synthesis during microbial interaction and proline analogue treatment drives the utilization for NADPH and provide NADP+, which is cofactor for G6PDH [22, 25]. So, it may improve cellular NADP^+^/NADPH ratio, which could stimulate G6PDH. As a result, deregulation of the pentose phosphate pathway may stimulate anabolism of erythrose-4-phosphate for biosynthesis of shikimate and phenylpropanoid metabolites [22, 26]. Meanwhile, proline acts as a reducing equivalent, in place of NADH to synthesize ATP through oxidation phosphorylation in the mitochondria [22, 27]. The relation of the enzymes with Nitrogen assimilation and the pentose phosphate pathway was shown in Fig. 1.

According to the correlation between the biosynthesis of exogenous lypsy phenolic.

substances and the reaction of plant antioxidant enzymes, a model of action of phytophenolmetabolites is proposed [22, 24, 28]. Through adopting more effective strategies, high nitrate concentration in water and soil also could produce similar reaction in plants and plants could tolerate stress. The early growth period is vital for any plant under nitrate stress, especially from germination to development of first two leaves. During early growth stages, nitrate assimilation of plants combined with proline-associated pentose phosphate pathway could provide a better defensive strategy against high nitrate concentrations. We predicted that the research on three feed-plant species including alfalfa (*Medicago sativa* L.), tall fescue (*Festuca arundinacea* L.) and perennial ryegrass (*Lolium perenne* L.) could clarify the relation of nitrate assimilation and proline-associated pentose phosphate pathway and mechanism of these feed-plant species to defend high nitrate concentrations.

The efficient utilization of absorbed nitrate in plants largely relies on the efficiency of nitrate reduction to ammonium and ammonium assimilation into amino acids, which are largely relevant to nitrate reductase activity. And photochemical efficiency has been chosen as light is important in the synthesis of nitrate reductase. Oxidation of carbohydrate through the oxidative pentose phosphate pathway gives reducing power and G6DPH is the regulatory enzyme in this procedure, so the G6DPH is a key factor in nitrite (NO2¯) reduction. In view that proline can scavenge reactive oxygen species as a reductant and proline-linked pentose phosphate pathway stimulates the generation of total soluble phenolics which plays an important role in countering oxidative stress, the proline content and total soluble phenolics content was measured in the study. SDH relate to TCA cycle which can produce NADH as reductant. The activity of key antioxidant enzymes such as SOD, CAT, GPX, can be stimulated by the proline under nitrate treatments. In the overall strategy for checking the efficiency that plants removing the nitrate in soil and ground water nitrate removal, we have measured total soluble phenolics content, nitrate reductase activity, G6PDH, proline content, SDH, activity of critical antioxidant enzymes and photochemical efficiency, then explored the relation of nitrate assimilation and proline-associated pentose phosphate pathway and mechanism of these feed-plant species defending high nitrate concentrations.

## Results

### Effect of nitrate concentration on germination percent

**Fig. 2:**
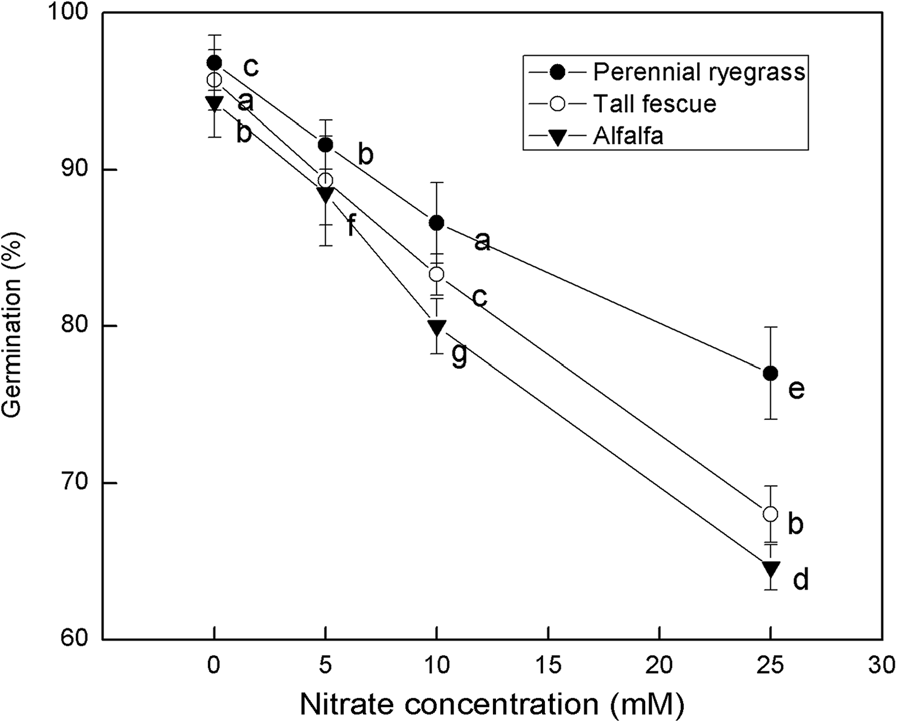
The germination percentage of three species at different nitrate concentrations. Means with different letters are significantly different (*p* < 0.05) showing treatment differences among three species

The germination rate of three kinds of feed-plant species treated with different concentrations of nitrates was shown in Fig. 2. At each concentration, the seeds of perennial ryegrass showed the highest germination percentage in lab condition while seeds of alfalfa had the lowest germination percentage. In the absence of nitrate treatment, the germination rate of the three feed plant seeds were all above 95%. The higher the concentration of nitrate is, the more obvious the difference in germination rates among the three plants. When the concentration of nitrate reached 25 mM, the difference of germination rate of the three plants was most obvious. At this time, the germination rate of alfalfa was the lowest, only 65%. But as a whole, with the increase of nitrate concentration, the germination rate of three feed plant seeds decreased significantly (*p* < 0.05).

### Total soluble phenolics and total antioxidant activity of three feed-plant species after seed nitrate treatments

**Fig. 3:**
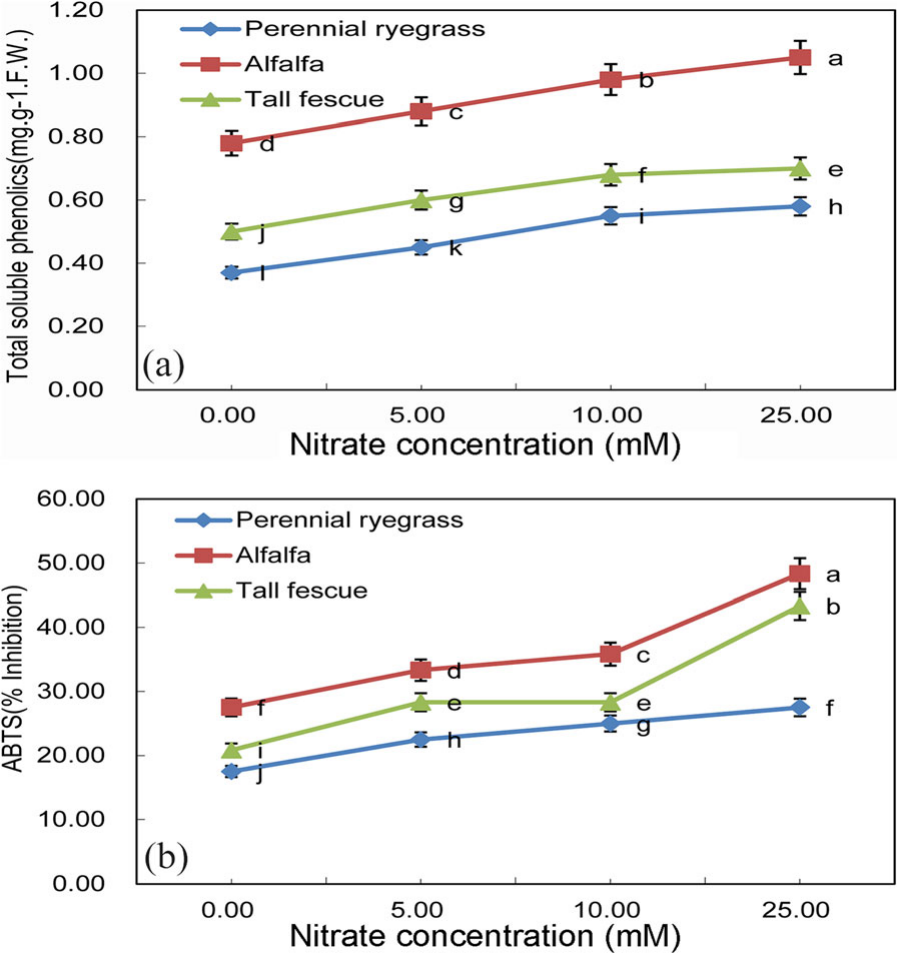
**a** Total soluble phenolic content (mg.g^− 1^ FW) and **b** Total antioxidant activity (%) of three feed-plant species (alfalfa, tall fescue and perennial ryegrass) after 2 weeks of germination with three seed nitrate treatments (5 mM KNO_3_, 10 mM KNO_3_ and 25 mM KNO_3_). Means with different letters are significantly different (*p*< 0.05) showing treatment differences among three species

Stimulation of phenolic biosynthesis was observed in all three feed-plant species after nitrate treatments when compared with control (Fig. 3a). There was a significant difference among three plant species (*p* < 0.05), and higher baseline total soluble phenolic content was seen in alfalfa, followed by tall fescue and perennial ryegrass. Under incremental concentrations of nitrate, total soluble phenolic content has largely increased (*p* < 0.05). The total soluble phenol content of Alfalfa increased from 0.80 mg.g^-1^.F.W. to 1.00 mg.g^-1^.F.W., an increase of 25%, while the content of perennial ryegrass’s total soluble phenol increased from 0.40 mg.g^-1^.F.W. to 0.50 mg.g^-1^.F.W. also increased by 25%. The antioxidant activity of plant shoots was carried out with the aid of free radical scavenging-linked ABTS assay. Similar to total soluble phenolic content, the free radical linked to antioxidant activities vary significantly between three plant species (*p* < 0.05), and highest was seen in alfalfa, followed by tall fescue and perennial ryegrass. From 5 mM to 10 mM KNO_3_ concentration, ABTS has little change. But from 10 mM to 25 mM KNO_3_, ABTS was significantly enhanced (*p* < 0.05) (Fig. 3b). Among them, the ABTS of alfalfa and tall fescue increased faster than that of perennial ryegrass. The ABTS of Alfalfa increased by 35%, tall fescue by 50%, and perennial ryegrass by little.

### Nitrate reductase, glucose-6-phosphate dehydrogenase and succinate dehydrogenase activity of three feed-plant species after seed nitrate treatment

**Fig. 4:**
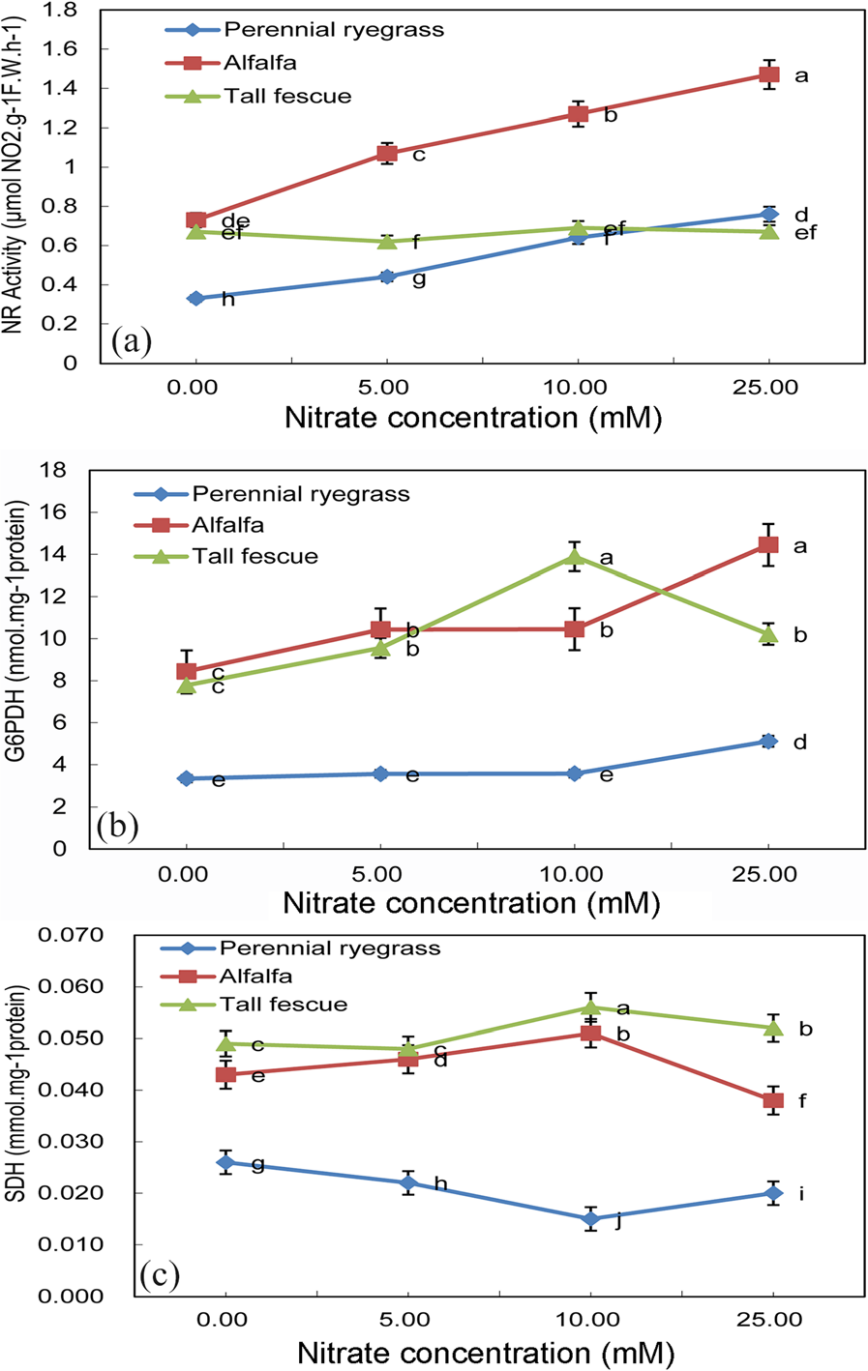
**a** Nitrate reductase activity (μmol NO_2_. g^− 1^ FW. h^− 1^), **b** Glucose-6-phosphate dehydrogenase activity (nmol.mg^− 1^ protein) and **c** Succinate dehydrogenase activity (mmol.mg^− 1^ protein) of three feed-plant species (alfalfa, tall fescue and perennial ryegrass) after 2 weeks of germination with three seed nitrate treatments (5 mM KNO_3_, 10 mM KNO_3_ and 25 mM KNO_3_). Means with different letters are significantly different (*p*< 0.05) showing treatment differences among three species

A nitrate reductase (NR) activity was observed in alfalfa, which was significantly higher than that of the other two plants, and increased gradually and significantly with the increase of nitrate concentration (*p* < 0.05), when the nitrate concentration was 5-25 mM, the NR activity of alfalfa was twice that of other two plants (Fig. 4a). The NR activity of perennial ryegrass showed a similar trend under nitrate treatment, but the highest activity was half of that of Alfalfa under 25 mM nitrate concentration. However, no significant change was observed in tall fescue, which was always around 0.6μmolNO_2_.g^-1^.F.W.h^-1^. The results showed that among these three plants, the concentration of nitrate had a greater effect on the activity of nitrate reductase of alfalfa and a little effect on the activity of nitrate reductase of tall fescue (*p* < 0.05). Glucose-6-phosphate dehydrogenase activity was also increased in all three feed-plant species under 25 mM KNO_3_ treatments (Fig. 4b). It was worth noting that the tendency of Glucose-6-phosphate dehydrogenase activity in tall fescue was much more different, which reached the peak at 10 mM KNO_3_ treatment, compared with the other two species. In contrast with perennial ryegrass, Alfalfa and tall fescue had markedly higher G6PDH content (*p* < 0.05). Another important enzyme succinate dehydrogenase in TCA (Kreb’s) cycle also showed a significant difference in these feed-plant species under varying nitrate treatments (Fig. 4c). Succinate dehydrogenase activity in perennial ryegrass was rather lower than the rest of two and in tall fescue is the highest, followed by Alfalfa (*p* < 0.05). With the increment of nitrate concentration, tall fescue and Alfalfa had a similar tendency which touched bottom at 10 mM KNO_3_ treatment, while perennial ryegrass was in a sharp contrast. Results of these three important enzymes indicate difference in pathway regulations among three different feed-plant species under nitrate treatments.

### Total proline content and proline dehydrogenase activity of three feed-plant species after seed nitrate treatments

**Fig. 5:**
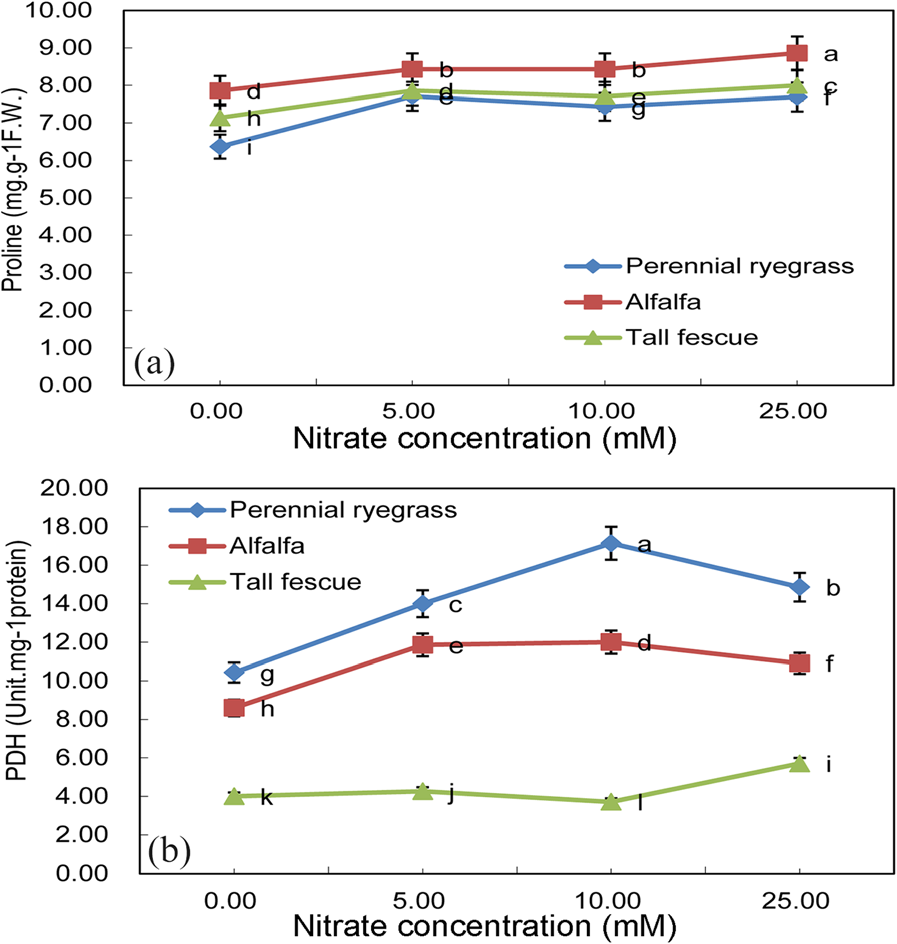
**a** Total proline content (mg. g^− 1^ FW) and **b** Proline dehydrogenase activity (Unit.mg^− 1^ protein) of three feed-plant species (alfalfa, tall fescue and perennial ryegrass) after 2 weeks of germination with three seed nitrate treatments (5 mM KNO_3_, 10 mM KNO_3_ and 25 mM KNO_3_). Means with different letters are significantly different (*p* < 0.05) showing treatment differences among three species

The total proline content of three plants under different nitrate treatment levels is shown in Fig. 5a. But there exists a significant difference among three plant species, and higher total proline content was seen in alfalfa, followed by tall fescue and perennial ryegrass, respectively(*p* < 0.05). Like total proline content and dehydrogenase activity was also significantly increased in perennial ryegrass under high nitrate treatments compared with control (Fig. 5b). The highest PDH activity was observed in perennial ryegrass under 10 mM KNO_3_ treatment, which was close to 17 unit.mg^-1^ protein. Different from total proline content, significantly higher PDH was seen in perennial ryegrass, followed by alfalfa and tall fescue, respectively(*p* < 0.05). And PDH activity in tall fescue changed slightly between 3unit.mg^-1^ protein-6 unit.mg^-1^ protein under different levels of KNO_3_ treatments.

### Superoxide dismutase, catalase and guaiacol peroxidase activity of three feed-plant species after seed nitrate treatments

**Fig. 6:**
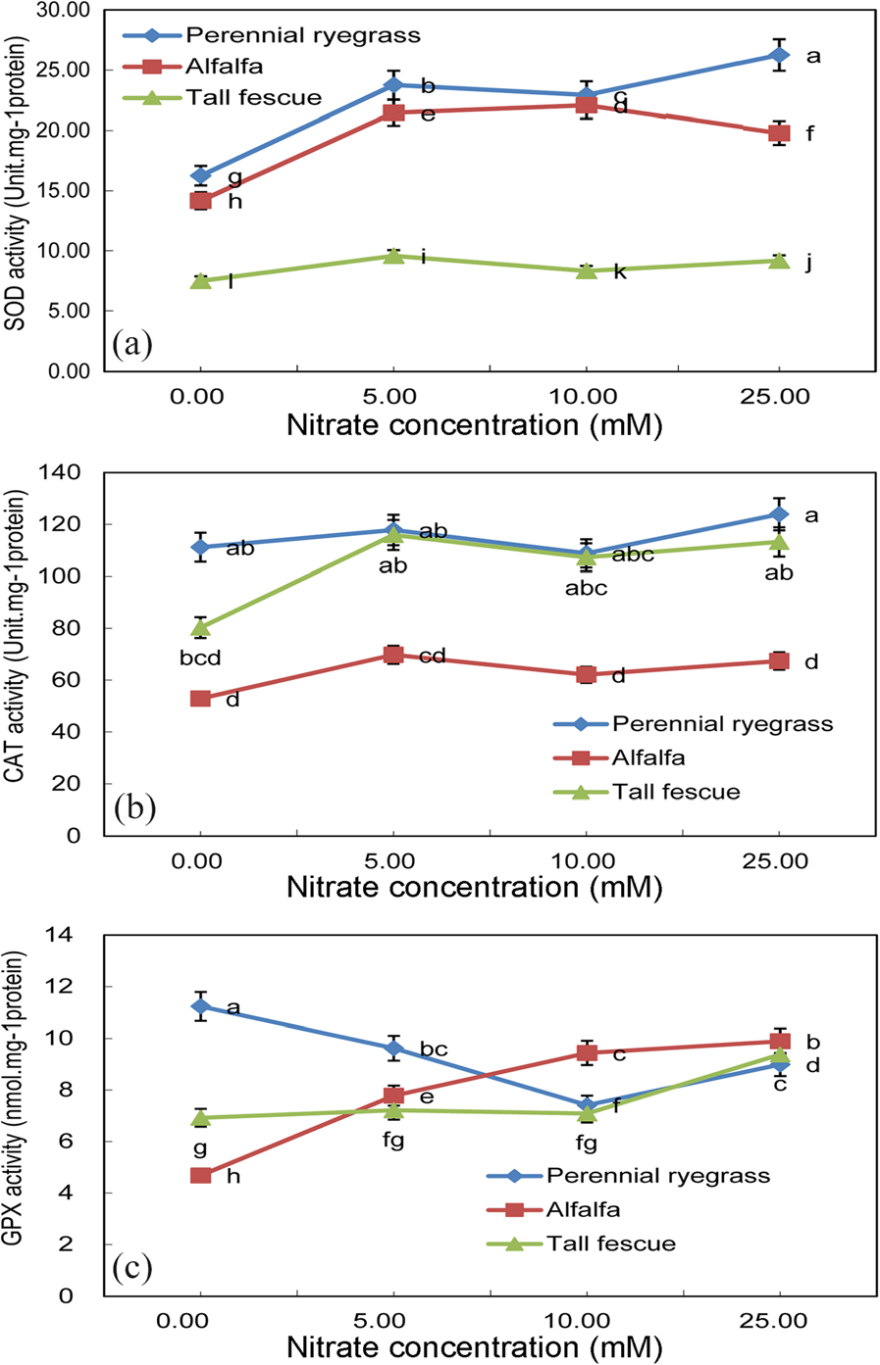
**a** Superoxide dismutase activity (Unit.mg^− 1^ protein), **b** Catalase activity (Unit.mg^− 1^ protein) and **c** Guaiacol peroxidase activity (nmol.mg^− 1^ protein) of three feed-plant species (alfalfa, tall fescue and perennial ryegrass) after 2 weeks of germination with three seed nitrate treatments (5 mM KNO_3_, 10 mM KNO_3_ and 25 mM KNO_3_). Means with different letters are significantly different (*p* < 0.05) showing treatment differences among three species

Different antioxidant enzymes were measured to determine the effect of nitrate treatments on antioxidant enzyme response system in these feed-plant species at early growth stages. Superoxide dismutase activity had a significant increase in perennial ryegrass and in alfalfa under higher nitrate treatments, while it did not change significantly in tall fescue (Fig. 6a). The SOD activity of perennial ryegrass was about 5 times that of tall fescue at 25 mM nitrate concentration. Higher superoxide dismutase activity in alfalfa and perennial ryegrass may be a counter measure to reactive oxygen species (ROS) in these two feed-plant species under high nitrate treatments. It can be seen from Fig. 6b that catalase activity in perennial ryegrass and tall fescue was rather higher than it in Alfalfa (*p* < 0.05). When the nitrate concentration was 5 mM, the catalase activity of perennial ryegrass increased by half, while the CAT activity of the other two plants did not increase as fast. After nitrate intake, the change of concentration had no significant effect on CAT activity of three plants (*p* > 0.05). The guaiacol peroxidase (GPX) activity in alfalfa significantly increased under nitrate treatment (*p*< 0.05) (Fig. 6c). When nitrate concentration changed from 0 mM to 10 mM, GPX activity of perennial ryegrass decreased significantly (*p*< 0.05), while the GPX activity of tall fescue increased not significantly (*p* > 0.05). However, when the nitrate concentration was 10 mM, the GPX activity of the two plants was almost the same, which was 7.5 nmol.mg^-1^ protein. However, the activity of GPX in three plants was significantly increased when the nitrate concentration changed from 10 mM to 25 mM (*p* < 0.05).

### Photochemical efficiency of three feed-plant species after seed nitrate treatments

**Fig. 7:**
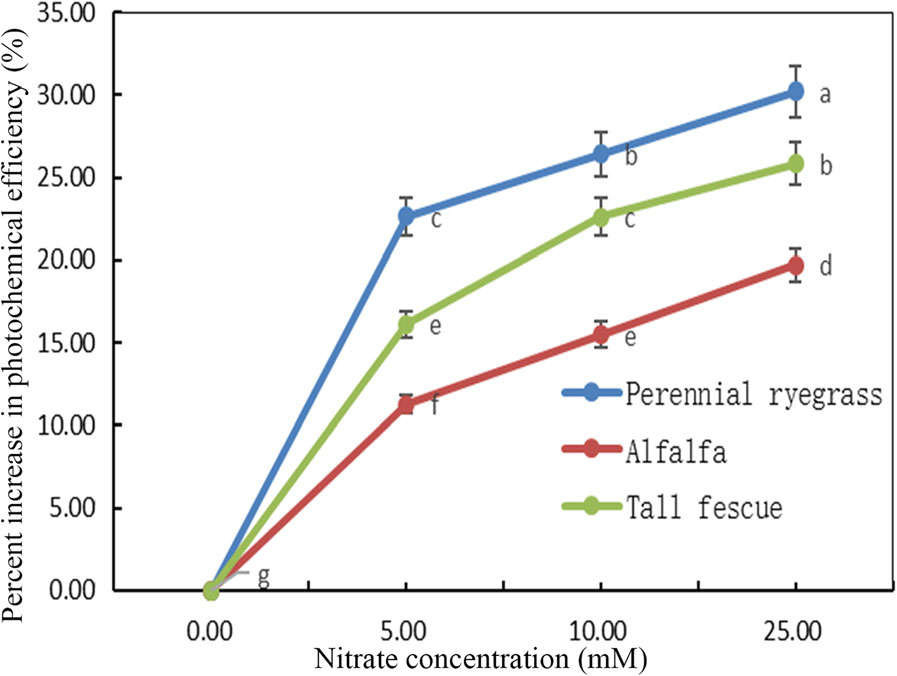
Percent increase in photochemical efficiency (%) of three feed-plant species (alfalfa, tall fescue and perennial ryegrass) after 2 weeks of germination with three seed nitrate treatments (5 mM KNO_3_, 10 mM KNO_3_ and 25 mM KNO_3_). Means with different letters are significantly different (*p* < 0.05) showing treatment differences among three species

Chlorophyll fluorescence (Fv/Fm) [29] can be used to indicate the physiological state of plant response to nitrogen stress. The photochemical efficiency of three feed-plant species was determined by using Fv/Fm technique and the increasing percentage of Fv / FM ratio was taken as the index. The increasing percentage of photochemical efficiency of three kinds of feed plants with different concentrations of nitrate treatment was significantly increased (*p* < 0.05) (Fig. 7). After nitrate treatment, the photochemical efficiency of perennial ryegrass increased the fastest and kept the highest growth efficiency. Higher photosynthetic activities in all three feed-plant species with seed nitrate treatment suggested that generation of NADPH and photosynthates in the leaves may contribute to reduction of the nitrate.

## Discussion

In the range of 0 mM to 25 mM nitrates, although the perennial ryegrass has a higher tolerance and the germination rate has decreased the least, on the whole, the germination rate of these three plants has decreased by about 25–30%, which showed a certain nitrate tolerance. This is consistent with the results obtained by Kołodziejek et al., who observed that a high concentration of potassium nitrate had negative effects on four kinds of dianthus seeds [30]. Figura et al. also observed that nitrate inhibited the germination of orchid seeds [31]. This may be because plant seeds are sensitive to nitrate concentration [32]. An increase in nitrate concentration may lead to toxic effects that inhibit seed germination, cell death, and loss of vigor, thereby reducing seed germination rate [33]. The germination rate can measure the growth of seeds and has a predictive effect. However, without measuring their growth, it is impossible to accurately know that these three plants are affected by nitrate during the later growth process, and no evidence of practical application can be obtained. In the future, we will also pay attention to using germination rate and growth together as indicators, making it convincing.

The phenomena of high total antioxidant activity in addition to higher total soluble phenolic content in alfalfa, tall fescue under high nitrate treatments, indicated that a possible accumulation of phenol with antioxidant response system in these species could counter cellular oxidative stress. Phenolic compounds are secondary metabolites extensively spread in plants [34], having the ability to reduce, stabilize, and dissociate unpaired electrons, reacting with other antioxidants, and transition metals chelate potentials, thus having antioxidant activity, which playing an important role in the defense path of plant antioxidant defense [24, 25, 35]. High total soluble phenolics accumulation in all three feed-plant species in high nitrate treatment suggested a possible mechanism of phenolic antioxidants against oxidative stress is either by means of a direct free radical scavenger, or through indirect stimulation of antioxidant enzymes response system [25]. Under abiotic stresses, both total soluble total phenolic content and total antioxidant activity in plants are higher [36, 37].

Nitrate reductase activity was also coupled with G6PDH activity both in alfalfa and in perennial ryegrass under high nitrate treatments. Results indicate that high G6PDH activity drives pentose phosphate pathway in these two species and generation of NADPH through the pentose phosphate pathway reduce nitrate. Saturation of nitrate reductase activity in tall fescue means a limitation of nitrate assimilation in this species in the early growth stage. Higher G6PDH activity with nitrate and nitrite was also observed in *Penicillium chrysogenum* [38] and in *Chlamydomonas reihardtii* [17] earlier. Different isoform of nitrate reductase may support pathways linked to pentose phosphate pathway where NADPH is generated and NADH from the TCA cycle can be replaced. In the research of Yinggao Liu et al. [39] and Hipkin et al. [40], a reasonable explanation can be seen, is that G6PDH under salt stress participates in the production of NR dependent NO and is therefore related to NR activity. Further conversion into NR and G6PDH has a strong relationship, which they are increasing or decreasing synchronously.

Perennial ryegrass showed high G6PDH activity and low SDH activity under high nitrate treatment. It may be due to the transfer of carbon flux from glycolysis to pentose phosphate pathway so that the NADPH decreased, and phenol content positively related to the pentose phosphate pathway related to proline correspondingly decreased [25]. But tall fescue showed different pathway regulations under high nitrate treatments with slightly increased G6PDH and SDH activity.

High proline content with high PDH activity in perennial ryegrass suggests a probable efficient proline oxidation in this species to sustain oxidative phosphorylation under high nitrate treatments. Higher nitrate treatment may promote glycolysis and pentose phosphate pathway to synthesize NADPH, thus improving germination rate, and may also induce proline synthesis [36], thus protecting nitrate reductase and proline oxidation in some species (such as perennial ryegrass), thus saving energy and playing a more effective role under pressure. This was also consistent with the fact that Sarkar et al. observed, they found that higher pentose phosphate pathway stimulation in perennial ryegrass [25]. Besides, Alfalfa and tall fescue may adopt different mechanism and pathway regulations to counter nitrate induced oxidative stress by generating more NADPH and supporting different anabolic need for cellular function.

Alfalfa countered nitrate-induced oxidative stress through higher activity of SOD and GPX, high activity of SOD and catalase was found in perennial ryegrass and tall fescue, respectively. This may be because the stimulation of SOD and elimination of CAT is changed by the change of individual phenols [25]. What’s more, high nitrate treatment caused that the excessive production of active oxygen made oxidative stress. Plant antioxidant enzymes including SOD, CAT, etc. will increase to scavenge reactive oxygen species against oxidation and maintain cell homeostasis [41, 42].

High photochemical efficiency along with high drive of the pentose phosphate pathway indicated that carbon flux may be a shift and utilize in different cellular mechanisms to meet cellular needs and to maintain redox balance through induction of different response systems. Hong et al., Wang et al., and Al Gehani et al. pointed out that nitrate can increase photosynthetic pigment and reduce the production of active oxygen, further induce the improvement of potential photochemical efficiency of PSII, and increase the electronic transport activity of PSII; nitrate may also unregulated antioxidant genes, stimulate the production of antioxidant enzymes, so as to improve the efficiency of F_v_ / F_m_ [43–45]。At the same time, nitrate was mediated by osmotic uptake of water and synthetic proteins, allowing the photosynthetic system to self-repair and enhance F_v_ / F_m_, affecting the positive development of plants [46–48]. The study of Al Gehani [49] and others pointed out that appropriate nitrate can reduce the salinity effect of salinized plants and promote plant growth. Therefore, it can be explained that the percentage increase in the photochemical efficiency of the three plants under nitrate treatment. In addition, it also can be seen in the study of Al Gehani et al. [49] that the salt tolerance of tomato seedlings can be improved by adding nitrogen levels of NO_3_NH_4_.

However, the experiment discusses a simple case, which is consistent with the situation that nitrate is not changed by the outside world. If the nitrate is migrated due to factors such as water erosion, the actual concentration will decrease, and the resulting experimental phenomenon will be closer to the experimental phenomenon produced by the lower concentration, which is complicated and difficult to determine. Therefore, research on the purification of nitrate by wetlands and/or aquatic plants may be the next step.

## Conclusion

The above results suggested that seeds of all three feed-plant species were able to tolerate and germinate properly at 25 mM KNO_3_ treatments. Among the three seeds, the germination rate of perennial ryegrass was the highest, followed by tall fescue, and the lowest was alfalfa, which was consistent with the initial germination rate without nitrate treatment. The initial growth and cellular function also remained normal under this treatment. The mechanism of initial tolerance and biochemical adjustments varied among three feed-plant species under nitrate treatments. Alfalfa was found more robust and adopted phenolic-linked induction of antioxidant response by driving pentose phosphate pathway coupled with nitrate reduction. G6DPH which was the first committed step of the pentose phosphate pathway suggests the increase of NADPH, therefore supported the reduction of nitrate to nitrite with the aid of nitrate reductase. With higher SOD in alfalfa and Perennial ryegrass indicated that the species counter reactive oxygen species through the induction of high antioxidant enzymes. Perennial ryegrass was also showed partly different efficient biochemical regulations to counter oxidative stress induced by nitrate during early growth stages. It can be speculated that critical antioxidase such as SOD, CAT, GPX, and proline-associated pentose phosphate pathway were likely to play an important role in tolerating the nitrate stress. Tall fescue did not respond the same way and might adopt a different mechanism to suffer high nitrate stress. That utilized proline-associated pentose phosphate pathway resulting in the stimulation of phenolic phytochemical in plants and CAT also helped. The high nitrate tolerance provided people an opportunity to use and cultivate these species in soil or water that contaminated by nitrate, which has helped to generate additional value as food and fodder.

## Methods

### Determination of seed germination rate

At the stage of seed germination, seeds (Yipin Company, Jiangsu, China) of alfalfa (*Medicago sativa* L.) variety- Algonquin, tall fescue (*Festuca arundinacea* L.) variety- Golden Island and perennial ryegrass (*Lolium perenne* L.) variety- Sun Island were collected as materials and sprouted under different nitrate concentrations. Representative voucher specimens of the studied material were deposited in the Wuhan Botanical Garden, Chinese Academy of Sciences. The collection of botanical material was performed under the direction of the biologist Yan Li. Seeds were treated in three different nitrate concentrations (5 mM KNO_3_, 10 mM KNO_3_ and 25 mM KNO_3_) and one control was performed with clean water and determined the seed germination rate. Shortly speaking, 25 alfalfa seeds and 50 tall fescue and 50 perennial ryegrass seeds were placed in conical flasks respectively, immersed with 250 mL of different concentrations of nitrate solutions, and then shook overnight by the shaking table. These seeds were then transferred to a petri dish with three layers of absorbent paper and one layer of Whatman # 1 filter paper moistened with a corresponding concentration of the nitrate solution. These dishes were then placed in a room at 20 °C along with continuous white light (340 μmol.m^-2^. s^-1^). Replace the old filter paper with Whatman # 1 filter paper wetted with nitrate solution of corresponding concentration every other day. Taking germ root to breakthrough half of the seed bark length as a germination standard, 1 week later, and the total number of germination was registered, the germination rate of the seeds was calculated, and samples of growing tissues were collected for biochemical analysis.

### Enzyme extraction

Refer to the experiment method of Lin et al. [24], configured enzyme extraction buffer—added 0.5% polyvinylpyrrolidone (PVP) and 3 mm EDTA to 0.1 m potassium phosphate buffer with a pH value of 7.5, and then used the cold pestle and motor to grind the plants leaf tissue (200 mg). After that, centrifuged at 12000×g at 2–5 °C for 15 min and then stored on ice [24, 50]. The supernatant was collected for analysis.

### Total protein assay

The means of Bradford assay was taken to measure protein content [21, 24]. Firstly, diluted the dye reagent concentrate (BioRad protein assay kit II, Bio-Rad Laboratory, Hercules, CA) with 4 times distilled water. Then, took 5 mL of diluted dye reagent and 100 μL of plant tissue extract to vortex and incubate for 5 min. Finally, used a UV-VIS Genesys spectrophotometer (Milton Roy, Inc., Rochester, NY) to measure the absorbance of the 5 mL reagent blank and 100 μL buffer solutions at 595 nm.

### Total soluble phenolics assay

The Folin-Ciocalteu method [24, 51] was used to analyze the total phenolic content in plant leaves. The absorbance was acquired at 725 nm. The absorbance readings were translated to total phenolics and were expressive of milligrams equivalents of gallic acid per grams fresh weight (FW) of the sample. Utilizing various concentrations of gallic acid in 95% ethanol established the standard curves*.*

### ABTS [2, 2′-azino-bis (3-ethylbenzthiazoline-6-sulphonic acid)] cation radical and antioxidant activity assay

The total antioxidant activity of creeping bent plant leaf extract was determined by the ABTS^+^ radical cation-decolorization assay involving performed ABTS^+^ radical cation [52]. The ABTS^+^ radical cation was prepared by the reaction of ABTS (Sigma Chemical Co.St. Louis, MO) aqueous solution and potassium persulfate, and then kept in the dark at room temperature for 12-16 h, and then analyzed.

Before analysis, diluted the ABTS^+^ stock solution with 95% ethanol (ratio 1:88), and.

obtained the absorbance at 734 nm of 0.70 ± 0.02, then balance to 30 °C. Add mL of ABTS volume to the glass tube containing 50 uL of each tissue extract, and mixed by vortex mixer for 30 s. Over 2.5 min incubation, the absorbance of mixtures was acquired at 734 nm. 5 mM stock solution of Trolox in ethanol was used to analyze, and the activity range of the assay within 0–20 μM final concentration. The percent inhibition was calculated by:

$$ \% inhibition=\frac{{A_{734}}^{control}-{A_{734}}^{extract}}{{A_{734}}^{control}}\times 100 $$

### Nitrate Reductase activity assay

Snell and Snell [53] described an assay to determine nitrate reductase (NR) activity of plants leaf tissue (1949) which we had a modification and applied it into research. Nitrite concentration was measured by spectrophotometrically at the wavelength of 530 nm. Various concentrations of sodium nitrite (0, 0.02, 0.10, 0.50 μmol/mL) solution with distilled water set up the standard curves. Nitrate reductase activity was measured and calculated as μmol nitrite produced g FW^− 1^ h^− 1^.

### Glucose-6-phosphate dehydrogenase (G6PDH) assay

In this assay a modified version of the assay described by Deutsch (1983) was followed [54]. The ratio of change in absorbance per minute could quantify the enzyme in the mixture by the extinction co-efficient of NADPH (6.22 mM^− 1^ cm^− 1^).

### Succinate dehydrogenase (SDH) assay

The activity of succinate dehydrogenase was assayed by a modified method described by Bregman [55]. The ratio of difference in absorbance per minute could quantify the enzyme in the mixture using the extinction co-efficient of DCPIP (19.1 mM^− 1^ cm^− 1^).

### HPLC analysis of proline

An agilent 1100 liquid chromatograph equipped with a diode array detector (DAD 1100) was used for high performance liquid chromatography (HPLC) analysis. The reverse phase Nucleosil C18, 250 nm × 4.6 mm was analytical column, and the filler particle size was 5 μm. The mobile phase of the elution extract sample was 20 mM potassium phosphate (pH 2.5 phosphate), the flow rate was 1 mL min^− 1^, and the detection wavelength was 210 nm. L-Proline (Sigma chemicals, St. Louis, MO) was used to calibrate the standard curve [56]. The amount of proline in the sample was expressed as mg of proline per milliliter and converted to mg g^− 1^ FW.

### Superoxide dismutase (SOD) assay

In a competitive inhibition assay, the reduction of nitro blue tetrazolium (NBT) to blue formazan was performed by using xanthine-xanthine oxidase-generated superoxide. The reduction of NBT at 560 nm indicated spectrophotometric assay of SOD activity [57]. One unit of SOD was regulated as the amount of protein that stops NBT from reduction to 50% of the maximum.

### Catalase (CAT) assay

The activity of catalase was taken from measurement using a method originally described by Beers and Sizer [58]. Determined the disappearance of peroxides by spectrophotometry. The difference in absorbance ΔA_240_/min from the initial (45 s) linear portion of the curve was calculated. One unit of catalase activity was defined as amount that decomposes one micromole of H_2_O_2_.

$$ \mathrm{Units}/\mathrm{mg}=\frac{\left({\varDelta \varDelta}_{240}/\min \right)\times 1000}{43.6\times mg\; enzyme/ mL\; of\kern0.17em reaction\kern0.17em mixture} $$

### Guaiacol peroxidase (GPX) assay

This test adopted a modified version of assay developed by Laloue et al. [59]. The ratio of variation in absorbance per minute was used to quantify the enzyme in the mixture using the extinction co-efficient of the oxidized product tetraguaiacol (26.6 mM^− 1^ cm^− 1^).

### Photochemical efficiency

Photochemical efficiency of plant shoots was measured by using OS1-FL (Fluorometer, Opti-Sciences, Inc., Tyngsboro, Mass). The test was performed in dark adapted mode and F_v_/F_m_ (F_v_/F_m_ = [F_m_ – F_o_]/ F_m_ the ratio of variable fluorescence to maximal fluorescence) ratio was calculated. Then calculate the percent increase. Plants were held in dark at least 2 h before the determination.

### Statistical analysis

All experiments were conducted with four replications. The effect of nitrate treatments on plant seeds was determined on the basis of the analysis of variance (ANOVA) of the Statistical Package for Social Science (SPSS 18.0 for windows, SPSS Inc., Chicago, IL, U.S.A.). Differences among nitrate treatment on three feed-plant species were determined according to the least significant difference (LSD) test at the 0.05 probability level.

## Data Availability

The raw data from all experiments as well as the material used in this manuscript can be obtained from the corresponding author upon reasonable request.

## Abbreviations

ABTS, 2: 2′-azino-bis (3-ethylbenzthiazoline-6-sulphonic acid)
CAT: Catalase
DAD: Diode array detector
EPA: The Environmental Protection Agency
G6PDH: Glucose-6-phosphate dehydrogenase
GPX: Guaiacol peroxidase
HPLC: High performance liquid chromatography
MCL: Maximum contaminant level
N: Nitrogen
NBT: Nitro blue tetrazolium
NH_4_^+^: Ammonium
NIR: Nitrite reductase
NO_2_¯: Nitrite
NO_3_¯: Nitrate
NR: Nitrate reductase
PVP: Polyvinylpyrrolidone
ROS: Reactive oxygen species
SDH: Succinate Dehydrogenase
SOD: Superoxide Dismutase
